# Over-Expression and Prognostic Significance of FATP5, as a New Biomarker, in Colorectal Carcinoma

**DOI:** 10.3389/fmolb.2021.770624

**Published:** 2022-01-27

**Authors:** Qi-Shun Geng, Mei-Jia Yang, Li-Feng Li, Zhi-Bo Shen, Long-Hao Wang, Yuan-Yuan Zheng, Wen-Hua Xue, Jie Zhao

**Affiliations:** ^1^ Department of Pharmacy, The First Affiliated Hospital of Zhengzhou University, Zhengzhou, China; ^2^ Internet Medical and System Applications of National Engineering Laboratory, The First Affiliated Hospital of Zhengzhou University, Zhengzhou, China

**Keywords:** colorectal carcinoma, biomarker, FATP5, cell cycle, survival

## Abstract

**Background:** Fatty acid transporters (FATPs) family play an important role in the uptake and metabolism regulation of long-chain fatty acids, which influence the occurrence and developing of multiple tumors. Fatty acid transporter 5(FATP5), a member of FATPs family, participates in fatty acid transport and lipid metabolism and is related to tumor development, whose mechanism in colorectal cancer (CRC) remains unclear.

**Methods:** In this study, we comprehensively utilized a range of relevant bioinformatic tools along with multiple databases to analyze the expression of FATPs family and investigate the biological function and prognostic value of FATP5 in CRC. Besides, cell proliferation and cell cycle distribution analysis, western blotting and immunohistochemistry (IHC) further validated the conclusion of bioinformatics analysis.

**Results:** FATP5 is the only member of FATPs family which was overexpressed in CRC. In the survival analysis based on the GSE39582 databases, the low expression of FATP5 predicts poor prognosis in CRC. Similar results were also observed in GSE17536, GSE28814 and TCGA colon cohorts. The potential function of DNA methylation regulated the abnormal expression of FATP5 in CRC. In addition, enrichment analysis indicated that FATP5 also participates in the regulation of cell cycle. Furthermore, Gene Set Enrichment Analysis (GSEA) showed a strong negative correlation between FATP5 and cell growth, implying that it may participate in regulating cancer cell proliferation by the regulation of cell cycle G2/M transition. At last, we identified that FATP5 was overexpressed in colorectal carcinoma tissues through immunohistochemistry staining, and played an important role in cell cycle by cell proliferation and cell cycle distribution analysis.

**Conclusion:** This study suggested that FATP5 was overexpression in colorectal carcinoma and predicted favorable prognosis, indicating it as a novel appealing prognostic marker for CRC.

## Introduction

Due to factors such as aging population, rapid population growth, and environmental pollution, cancer incidence and mortality continued to increase worldwide. Among all cancers, the morbidity and mortality of CRC were at the forefront of global cancer statistics, which indicate it ranks the third among the most frequently diagnosed cancers and that it’s the fourth leading cause of cancer death worldwide (Favoriti et al., 2016; Bray et al., 2018). With the application of diagnostic technology and equipment, screening can effectively reduce the incidence and mortality of CRC. However, due to economic conditions and social status, CRC screening was only available to a small part of the global population, which led to wide variations in the morbidity and mortality of CRC (Schreuders et al., 2015). Due to changes in multiple system such as blood and immunity, the pathogenesis of colorectal cancer was very complicated (Mlecnik et al., 2016). Studies focusing on the molecular mechanism of CRC occurrence and development can be beneficial to the diagnose and treatment of this malignant tumor. However, the overall 5-years survival rate for patients with CRC is still very low (Overman et al., 2017). Therefore, finding reliable biomarkers for early diagnosis, accurate prognosis and replacement therapy still wait for further discovery.

Long-chain fatty acids (LCFA) are one of the main energy sources for cells, whose uptake and activation can affect many cellular processes, including membrane synthesis, intracellular signal transduction, and energy metabolism. The uptake of LCFA involves a variety of membrane proteins, such as FATPs (Stahl, 2004). The FATPs family consists of six members, FATP1-6, encoded by SLC27A1-6. FATPs are complete membrane proteins with at least one transmembrane domain, ranging in size from 63 to 80 KDa. The FATPs are also an indispensable transmembrane protein, which can enhance the absorption of long-chain and ultra-long-chain fatty acids to cells. All FATP members are located at the C-terminus, and have highly conserved 311 amino acid natural sequence, called FATP sequence, and AMP binding domain, which are responsible for LCFA binding and uptake. Despite the sequence similarity of FATP members, the proteins are differentially expressed in tissues and cell types (involving adipose tissue, liver, intestine, and endothelial cells, etc.) (Anderson and Stahl, 2013). FATP5 is a liver-specific member of the FATP family. The molecule of FATP5 is bile BAL, which catalyzes the binding of bile acids to amino acids and plays an unexpected part in bile acid metabolism. Previous studies indicated that FATP5 plays an important role in fatty acid transport, lipid metabolism, and weight regulation (Hubbard et al., 2006). Nevertheless, what role FATP5 plays in the progression of CRC is still ambiguous and remains to be elucidated.

**FIGURE 1:**
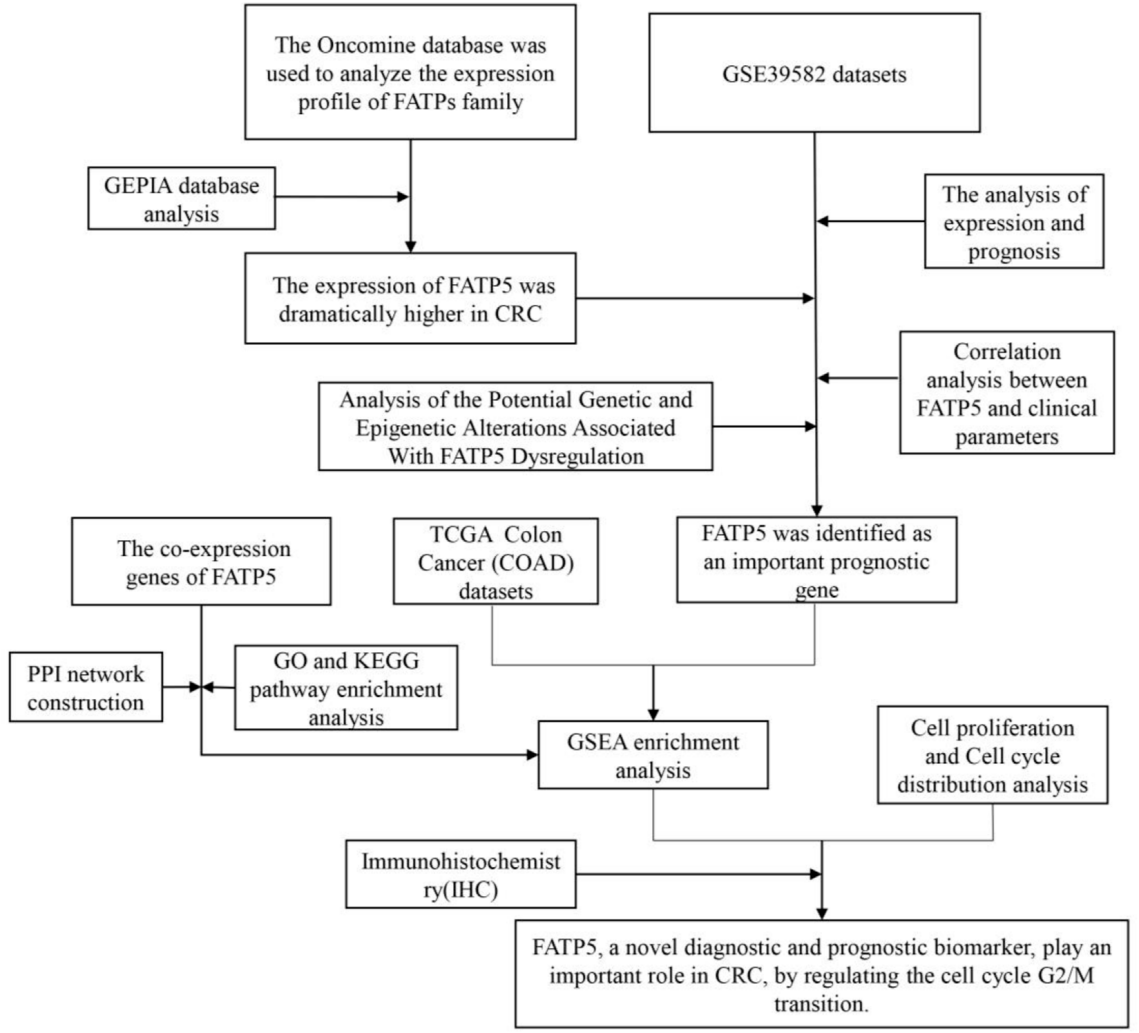
Flow diagram of the study.

In this study, we utilized a wide range of comprehensive bioinformatics methods and molecular biology experiments to analyze FATP5 expression, potential function, and impacts on prognosis of human CRC, which provided a theoretical basis for future clinical research. The study identified the overexpression of FATP5 in colorectal cancer tissues by immunohistochemical staining and played an important role in cell cycle by cell proliferation and cell cycle distribution analysis (Figure 1).

## Materials and Methods

### Cell Culture and Cell Transfection

CRC cells including CACO-2 and HCT116 cells were acquired from Chinese Academy of Sciences (Shanghai, China). The cells were maintained in DMEM with 10% FBS (Thermo, United States) and cultured at 37°C, 5% CO2 and saturated humidity.

FATP5-siRNAs (siFATP5) and control-siRNA (siNC) were chemically synthesized by Tsingke Biotechnology Co., Ltd (Beiing, China), and used for transfection into CRC cell lines. To silence the expression of FATP5, siRNAs specifically targeting to FATP5 was obtained. The target sequences of FATP5 is shown below: si-FATP5: 5-CAG​AAG​GCA​ACA​UGG​GCU​UAG​UCA​A-3. JetPRIME^®^ Transfection Reagent was used for cell transfection (Polyplus Transfection, United States).

### The Cancer Genome Atlas (TCGA) Database and Gene Expression Omnibus (GEO) Datasets

Gene expression array in CRC tumor and related clinical information were downloaded from the GEO2. 566 cancer samples and 19 normal samples were obtained from GSE39582 (557 samples had prognostic information and 525 samples had clinical parameters). In addition, the expression profiling of CRC patients and survival information were extracted from GEO: GSE17536 (*N* = 177) and GSE28814 (*N* = 125) datasets. The acquisition of FATP5 transcriptome data from colorectal tumors in the TCGA dataset is available from the Cancer Genome Browser website of the University of California, Santa Cruz (http://xena.ucsc.edu/) (Goldman et al., 2019), which was also used to elevate the correlation of FATP5 and cell cycle regulation. We retrieved CRC tumor sample (*n* = 285) data from TCGA.

### The Oncomine Platform and GEPIA Dataset

The Oncomine platform (https://www.oncomine.org/resource/login.html) providing open access to cancer microarray database online was used to facilitate the analysis of genome-wide expression in various cancers (Rhodes et al., 2004). We used the Oncomine database to compare the mRNA expressions of FATPs between clinical colorectal cancer specimens and normal controls, whose purpose was to pick out genes that are significantly differently expressed in FATPs. In order to obtain the important gene probe, we set the following thresholds: *p*-value < 1e-4, fold change >2 and the gene ranks in the top 10%. The online database Gene Expression Profiling Interactive Analysis (GEPIA) (http://gepia.cancer-pku.cn/index.html), as a newly developed interactive web server, enables RNA sequencing expression data of 9,736 tumors as well as 8,587 normal samples from the TCGA and GTEx projects (Tang et al., 2017). The gene expression of FATPs was verified in the GEPIA database, which was also used to gauge the expression and overall survival of genes.

### cBioPortal Database and Catalogue of Somatic Mutations in Cancer (COSMIC) Dataset

The cBioPortal (http://cbioportal.org) Cancer Genomics portal serves as a resource offering open data to interactively explore multidimensional genomic datasets on cancer and currently has access to over 5,000 tumor samples from 20 cancer studies, which can greatly assist researchers to access molecular profiles and clinical attributes in extensive cancer genome projects, and enable scientific research personnel to transform abundant data sets into relevant biological information and clinical applications (Cerami et al., 2012; Gao et al., 2013). We applied this database to determine the genes that are positively associated with FATP5 expression in CRC and obtained related information of copy number alterations (CNAs). COSMIC(http://cancer.sanger.ac.uk)—the Catalogue of Somatic Mutations in Cancer—is the world’s largest source of professional manually-compiled somatic mutation information relating to human cancers (Tate et al., 2019). This database compiles data, which covers a wide range of the field of cancer genomics in terms of somatic mutations. Therefore, we used the database to analyze mutations of FATP5 in CRC.

### DNA Methylation Analysis

MEXPRESS(https://mexpress.be/) is a data visualization tool meant to simplify the visualization of TCGA expression, DNA methylation and clinical data, along with the relationships between them (Koch et al., 2019), which was used to complete data analysis and get relevant data. Pearson’s correlation between FATP5 gene expression level and methylation of its CpG islands was evaluated by using R software.

### The PPI (Protein-Protein Interact) Network Diagram Construction and Function Enrichment Analysis of Co-Expressed Genes

The String (http://string-db.org/) (Szklarczyk et al., 2019) database is a system that searches for interactions between proteins. We select multiple protein tools and define the species as human, obtain a PPI network of co-expressed genes, which was visualized by Cytoscape (version 3.7.2) software (Smoot et al., 2011). ClueGO (version 3.0.3) (Maere et al., 2005) is a plug-in playing the character to visualize the nonredundant biological terms for large clusters of genes in a functionally-grouped network, which executed the biologic process of functional annotation analysis of co-expressed genes as well. DAVID Bioinformatics Resources (http://david.abcc.ncifcrf.gov/),a comprehensive biological biology and analysis tool that systematically extracts biological significance from gene or protein lists, was used for function enrichment analysis (Huang et al., 2009a; Huang et al., 2009b). Gene Set Enrichment Analysis is a computational method employed to determine whether a priori defined set of genes shows statistically significant, consistent difference between two biological states. Each sample was defined as either “H” or “L”, depending on whether it was greater than the median mRNA expression value of FATP5. GSEA was performed using clusterProfiler package with gene set c5 (c5. go.v7.4. symbols.gmt).

### Cell Proliferation and Cell Cycle Distribution Analysis

CCK-8 assays were performed by a CCK-8 kit from Beyotime (Beijing, China). In brief, cells in log phase growth were planted in 96-well dishes at a concentration of 5 × 10^3^ cells/well and cultivated in DMEM containing 10% FBS for cell attachment. Cell viability was detected with CCK-8 reagent followed by the protocol of manufacturer at the indicated time point (24, 48 and 72 h).

Propidium iodide (PI; Sigma, P4170) staining was used to analyze DNA content and cell cycle profile. Assays were performed 48 h after the transfection of siNC and siFATP5. Cells were washed twice with ice-cold PBS, harvested, and fixed with 70% ethanol at 4°C overnight. Cells were resuspended in PBS, permeabilized with 0.125% Triton X-100 (Sigma-Aldrich, T9284) and stained with 40 μg/ml PI for 30 min on ice. Cell cycle distributions were analyzed by flow cytometry (ACEA NovoCyteF, Agilent).

### qRT-PCR, Western Blotting and Immunohistochemistry(IHC)

TRIzol reagent (Invitrogen, Carlsbad, CA, United States) was used to extracted RNA from cells. Complementary DNA (cDNA) was synthesized from total RNA using PrimeScript RT reagent Kit (Takara, Dalian, China), and PCR was performed using SYBR Green RT-PCR Kit (AG11701, ACCURATE BIOTECHNOLOGY, HUNAN,Co.,Ltd.). GAPDH served as an internal control. PCR was run on the StepOne Plus Real-Time PCR System (Applied Biosystems, Foster City, CA, United States), and data were analyzed using the 2-ΔΔCT method. The primer sequences used in the experiment is as follow. FN1, 5′- GCT​CCC​TGC​CTA​TGC​CAC​ACC-3′ (forward), 5′- CAC​ACA​CAG​CCT​GGT​ACA​CAT-3′ (reverse).

Cells were lysed using RIPA buffer (Beyotime) supplemented with protease inhibitor cocktail (Thermo Scientific, Waltham, MA, United States) and PMSF. A BCA method was used to measure the protein concentration. 40 μg cell lysates were subjected to sodium dodecyl sulfate-polyacrylamide gel electrophoresis (SDS-PAGE), transferred to polyvinyldene fluoride (PVDF) membrane (Sigma, St Louis, MO, United StatesA). The membrane was incubated with specific antibody for FATP5 (1:1,000 dilution;Affinity, DF3845), p27 (1:1,000 dilution; Affinity, AF6324) and CCNB1 (1:2000 dilution; proteintech, 67686-1-Ig), followed by incubation with horseradish peroxidase-conjugated anti-IgG. Autoradiograms were densitometrically quantified (Quantity One software; Bio-Rad), with Β-actin serving as internal control.

Immunohistochemistry of colorectal cancer arrays (HCol-Can060 PT-01, SHANGHAI OUTDO BIOTECH CO,LTD.; National Engineering Center for Biochip at Shanghai; National Human Genetic Resources Sharing Service Platform, 2005DKA21300, China) was performed by using FATP5 antibody (1:200 dilution; Affinity). Briefly, after xylol deparaffinization and rehydration in graded alcohols, colorectal cancer arrays were boiled in citrate buffer, pre-incubated with H_2_O_2_, and blocked with rabbit or goat serum (DAKO, Glostrup, Denmark). Arrays were then incubated with a primary antibody and then with an HRP-conjugated secondary antibody. Before counterstaining with hematoxylin, diaminobenzidine was used to develop the target proteins. The IHC staining was evaluated by applying a scoring system from 0 to 3 (0 = negative or no staining, 1 = weak or low staining; 2 = moderate or intermediate staining; and 3 = strong or high staining), which determine the score for each tissue based on the percentage of positive cells and intensity of staining. The arrays were read by a pathologist using Olympus BX41 microscope.

### Statistical Analysis

By default, all statistical analysis is performed as described in the web resources. In short, the Student’s *t*-test is used to compare the expression of mRNA in the Oncomine database. GEPIA difference analysis uses one-way ANOVA by defining disease status (tumor or normal) as a variable. Spearman correlation analysis was used to evaluate the correlation of gene expression in cBioPortal and UCSC databases. Fisher’s exact test is used to measure gene richness in annotated terms in David’s annotation system. Besides, the χ^2^ test was utilized for analysis on correlation between FATP5 expression and clinical parameters. Univariate and multivariate analyses were performed using Cox proportional hazard model. *p* < 0.05 indicates statistical significance (*, *p* < 0.05; **, *p* < 0.01).

## Results

### Overexpression of FATP5 in Colorectal Cancer

**FIGURE 2:**
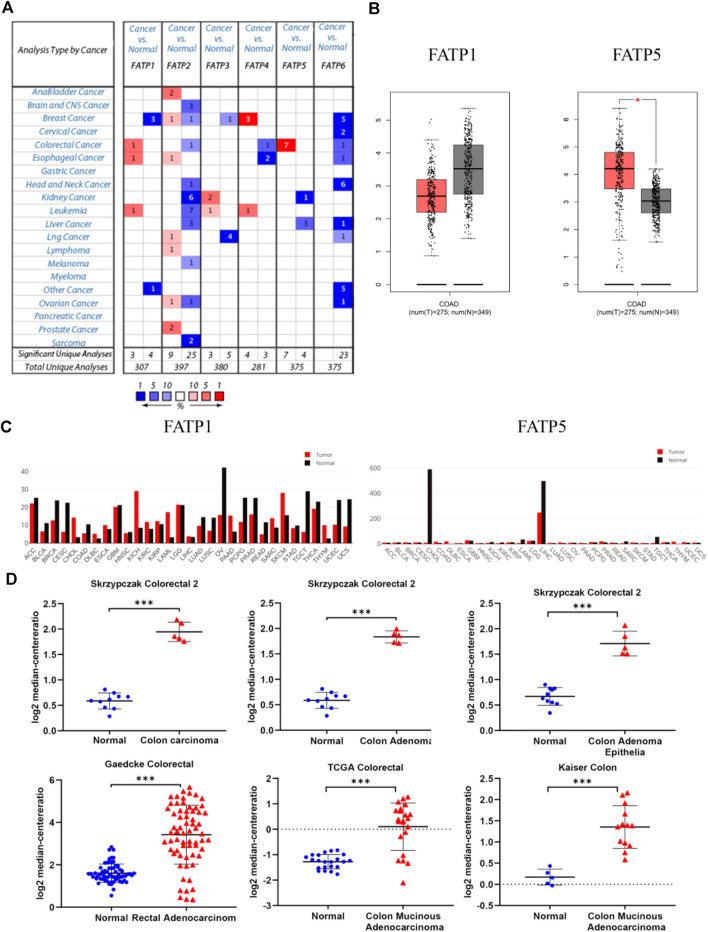
FATP5 mRNA expression was elevated in human colorectal cancer (CRC). **(A)** Using the Oncomine database, this graph shows the numbers of datasets with statistically significant mRNA overexpression (red) or downexpression (blue) of FATPs (cancer tissues vs corresponding normal tissues). The threshold was defined with the following parameters: *p*-value of 1E-4, fold change of 2 and gene ranking of 10%. **(B–C)** The GEPIA database verified that FATP5 gene expression was significantly upregulated in colon cancerFATP1 tissues (*n* = 275) compared with normal colon tissues (*n* = 349), *p < 0.05. **(D)** The Oncomine database analysis of FATP5 gene expressions in different subtypes of colorectal cancer, ***p < 0.001.

The Oncomine database was used to analyze the expression profile of FATPs family. We found that the expression of FATP1 and FATP5 was significantly elevated in colorectal cancer (Figure 2A). Then, we determined the expression of FATP1 and FATP5 using GEPIA database, which further validated FATP5 was the only member of FATPs family that upregulated in colorectal cancer tissues compared to normal (Figures 2B,C). What’s more, Oncomine analysis of cancer vs normal samples in different patient datasets showed that the expression of FATP5 was dramatically higher in colon adenoma, colon carcinoma epithelia, colon mucinous adenocarcinoma and rectal adenocarcinoma (Figure 2D; Table 1).

**TABLE 1:** The Oncomine database analysis indicated that FATP5 expressions are upregulated in different subtypes of colorectal carcinoma.

Types of esophageal cancer vs. normal	Fold change	*p* Value	*t*-test	Ref
TCGA colorectal				
Rectal mucinous adenocarcinoma vs. Normal	3.406	2.81E-9	11.057	TCGA
Rectosigmoid adenocarcinoma vs. Normal	3.917	4.54E-8	13.404	TCGA
Colon mucinous adenocarcinoma vs. Normal	2.568	9.67E-11	8.347	TCGA
Cecum adenocarcinoma vs. Normal	2.715	5.25E-11	8.571	TCGA
Rectal adenocarcinoma vs. Normal	2.520	7.81E-13	10.204	TCGA
Colon adenocarcinoma vs. Normal	2.563	7.23E-13	10.840	TCGA
Sabates-Bellver Colon				
Colon adenoma vs. Normal	3.588	5.92E-16	11.830	Sabates-bellver
Rectal adenoma vs. Normal	4.886	1.81E-6	10.286	Sabates-bellver
Gaedcke colorectal				
Rectal adenocarcinoma vs. Normal	3.141	2.06E-30	15.701	Gaedcke
Skrzypczak colorectal				
Colorectal carcinoma vs. Normal	2.965	1.06E-7	6.334	Skrzypczak
Hong colorectal				
Colorectal carcinoma vs. Normal	2.783	5.36E-9	9.134	Hong
Skrzypczak colorectal 2				
Colon adenoma vs. Normal	3.558	3.13E-5	6.224	Skrzypczak

### Low FATP5 Expression Predicts Poor Prognosis in Colorectal Cancer

**FIGURE 3:**
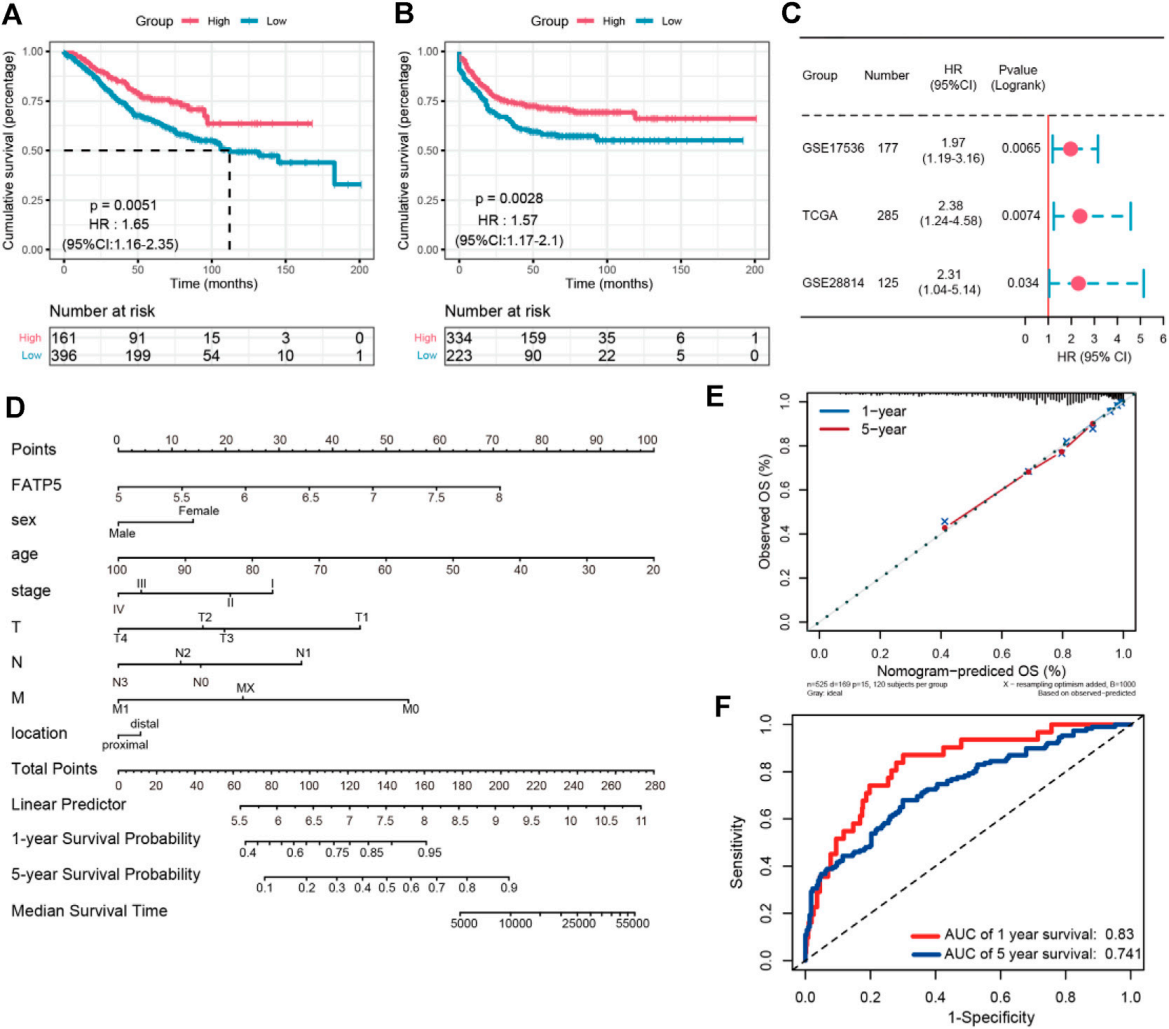
The survival analysis of FATP5 in human colorectal cancer. The overall survival **(A)** and relapse free survival **(B)** of FATP5 in colorectal cancer based on GSE39582 accession. **(C)** The survival analysis of FATP5 in GSE17536, GSE28814 and TCGA colon cohorts. **(D)** A nomogram consisting of risk score and other clinical indicators for predicting 1-, and 5-years OS of CRC. **(E)** Calibration plot of nomogram for predicting probabilities of 1, and 5-years overall survival of patients. **(F)** Time-dependent ROC for 1-, and 5-years OS predictions for the nomogram compared with actual observations.

χ-tile 3.6.1 software was used to identify the optimum cut-off value for distinguishing high-expression FATP5 patients from low-expression FATP5 patients. Low-expression FATP5 patients had shorter overall survival (OS) (HR = 1.65; 95% CI: 0.216–2.35; *p* < 0.01) and shorter relapse free survival (RES) (HR = 1.57; 95% CI: 1.17–2.1; *p* < 0.01) than patients with high-expression FATP5 patients (Figures 3A,B). To determine whether the FATP5 expression for predicting prognostic was robust, the performance of the FATP5 expression was assessed in three independent colon cohorts, which totally consisted of 587 colon patients. The optimum cut-off value for colon cohorts was identified with χ-tile 3.6.1 software, based on the expression of FATP5. Similar results were observed in GSE17536, GSE28814 and TCGA colon cohorts, which suggested that low-expression FATP5 group had significantly worse OS than those who were assigned to the low-expression FATP5 group according to the expression of FATP5 (Figure 3C). We further compared the clinical characteristics (including age, sex, location, clinical stage, and tumor grade) between FATP5 expression-high and low groups and managed to observe obvious statistics differences in lymph node metastasis and distant metastasis, while no observation for other clinical features indicated significant differences (Table 2). In addition, the univariate and multivariate Cox regression model using GSE39582 accession demonstrated that distant metastasis and FATP5 expression were irrelevant prognostic factors for OS of patients with CRC (Table 3). In order to further illustrate the importance of FATP5 for prognosis, we construct a nomogram based on clinical parameters and the expression of FATP5 (Figure 3D). The ROC and the calibration curves indicated decent accuracy (Area Under Curve (AUC) of 1-year survival: 0.828; AUC of 5-years survival: 0.738) and good discrimination (C-index = 0.763) (Figures 3E,F).

**TABLE 2:** Comparison of clinical characteristics between low-expression FATP5 group and high-expression FATP5 group in GSE39582 cohort.

Variable	Case NO. (%)	FATP5		*p*
		High	Low	
Sample	525	262	263	—
Age(Year)				
≥60	380	189	191	0.901
<60	145	73	72	
Gender				
Female	240	120	120	0.968
Male	285	142	143	
Clinical stage				
I	31	19	12	0.001
II	247	135	112	
III	189	92	97	
IV	58	16	42	
Tumor stage				
T1	11	6	5	0.075
T2	43	27	16	
T3	356	182	174	
T4	115	47	68	
Lymph node metastasis				
Yes	235	104	131	0.020
No	290	158	132	
Distant metastases				
Yes	61	16	45	<0.001
No	464	246	218	
Location				
Proximal	208	106	102	0.695
Distal	317	156	161	

**TABLE 3:** Univariate and multivariate regression analyses for predicting overall survival in GSE39582 cohort.

Variable	Univariate			Multivariate		
	HR	(95% CI)	*p*	HR	(95% CI)	*p*
Age (ref.<60)	1.40	0.95 ∼ 1.90	0.098	1.47	1.02 ∼ 2.12	0.04
Gender (ref. Male)	0.77	0.57 ∼ 1.00	0.093	0.75	0.55 ∼ 1.02	0.069
AJCC stage (ref. I-II)	2.00	1.40 ∼ 2.70	<0.001	1.56	0.66 ∼ 3.67	0.307
pT stage (ref. T1-T2)	1.90	0.95 ∼ 3.70	0.068	1.41	0.71 ∼ 2.80	0.323
pN stage (ref. N0)	1.70	1.20 ∼ 2.30	<0.001	0.91	0.41 ∼ 2.01	0.824
pM stage (ref. M0)	5.50	3.80 ∼ 7.90	<0.001	4.41	2.85 ∼ 6.82	<0.001
Location (ref. Proximal)	0.89	0.65 ∼ 1.20	0.45	0.85	0.62 ∼ 1.17	0.313
FATP5 expression (ref. low)	0.62	0.45 ∼ 0.84	0.002	0.69	0.23 ∼ 0.99	0.017

### Analysis of the Potential Genetic and Epigenetic Alterations Associated With FATP5 Dysregulation

**FIGURE 4:**
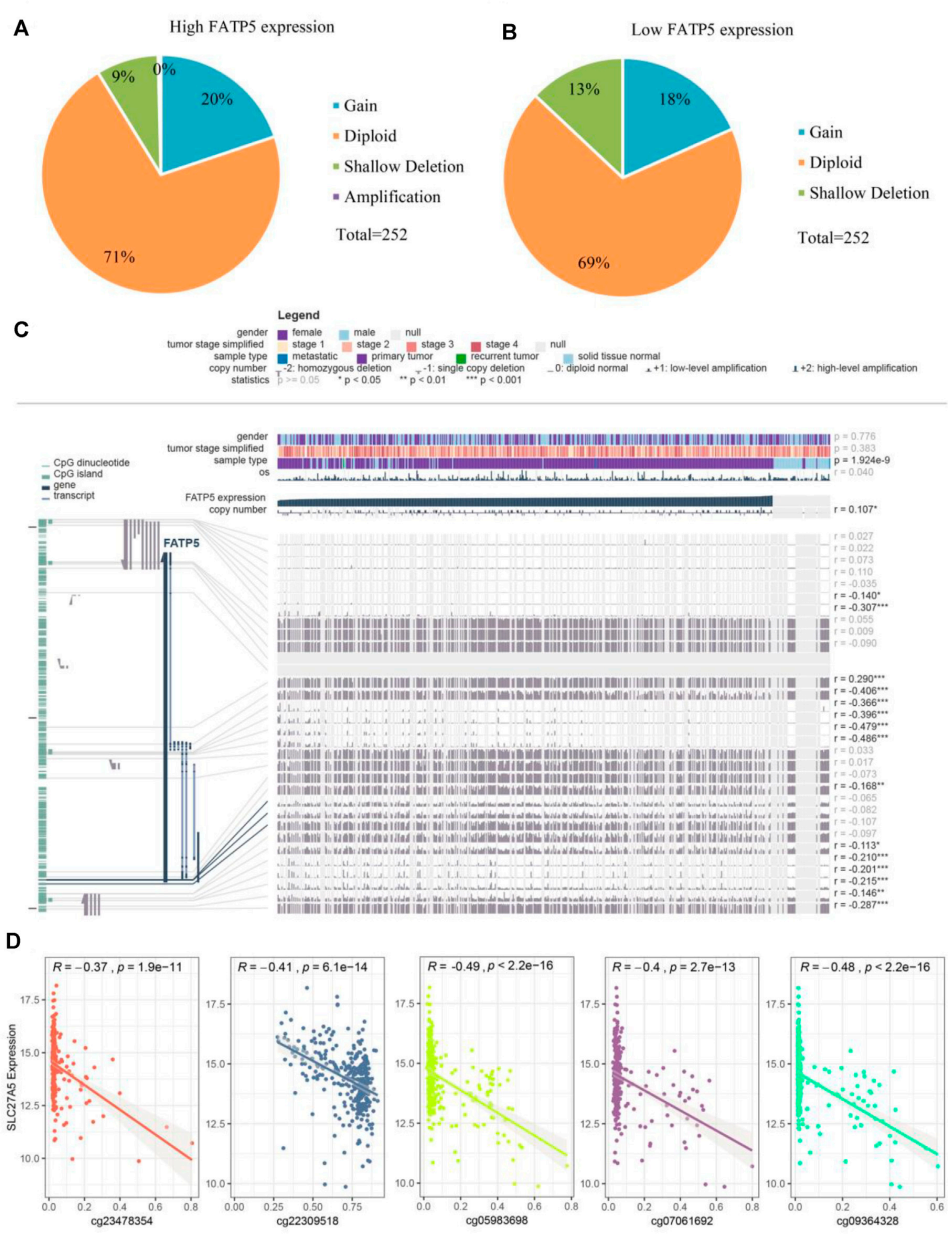
Analysis of the potential genetic and epigenetic alterations associated with FATP5 dysregulation. **(A,B)** The CNAs in FATP5-high and low expression groups. **(C)** Analysis of CpG island methylation and abnormal FATP5 expression using TCGA dataset. **(D)** Correlation between FATP5 expression and CpG island methylation was performed. ***p < 0.001.

Considering rare mutation and significant prognosis of FATP5, we next explored the potential mechanism of FATP5 abnormal expression in CRC. Copy number alterations (CNAs) has been confirmed as a fundamental mechanism of oncogene activation in tumor (Chang et al., 2020). Aimed to ascertain whether there is firm evidence to establish the casual association between CNAs and the abnormal expression of FATP5 in CRC, we analyzed data with CNAs available in TCGA dataset by using CbioPortal tool. The patterns of CNAs were no significant differences in the FATP5-high and low groups (Figures 4A,B). Analogous to CNAs’ activating oncogene function, studies integrating DNA-methylation profiles and gene expression data have testified that methylation in different genomic regions is related to gene expression levels (Moore et al., 2013). To investigate whether DNA methylation results in FATP5 dysfunction, MEXPRESS was used to examine the status of CpG sites in colorectal carcinoma (Figure 4C). There are 15 CpG sites with a significant correlation with the expression of FATP5 (*p* < 0.05); among them, 14 CpG sites, including cg23478354, cg22309518, cg07061962, cg05983698, cg09364328 etc., were negatively correlated with FATP5 expression. Then, we employed the Pearson correlation coefficient to the five CpG sites with the greatest correlation and found that FATP5 expression was negatively correlated with the five CpG sites (Figure 4D, *p* < 0.001). Collectively, these data demonstrated the potential function of DNA methylation in regulating the abnormal expression of FATP5 in CRC, showing the potential mechanism is worth further exploring.

### PPI Network Construction and Analysis of Co-expression Genes

**FIGURE 5:**
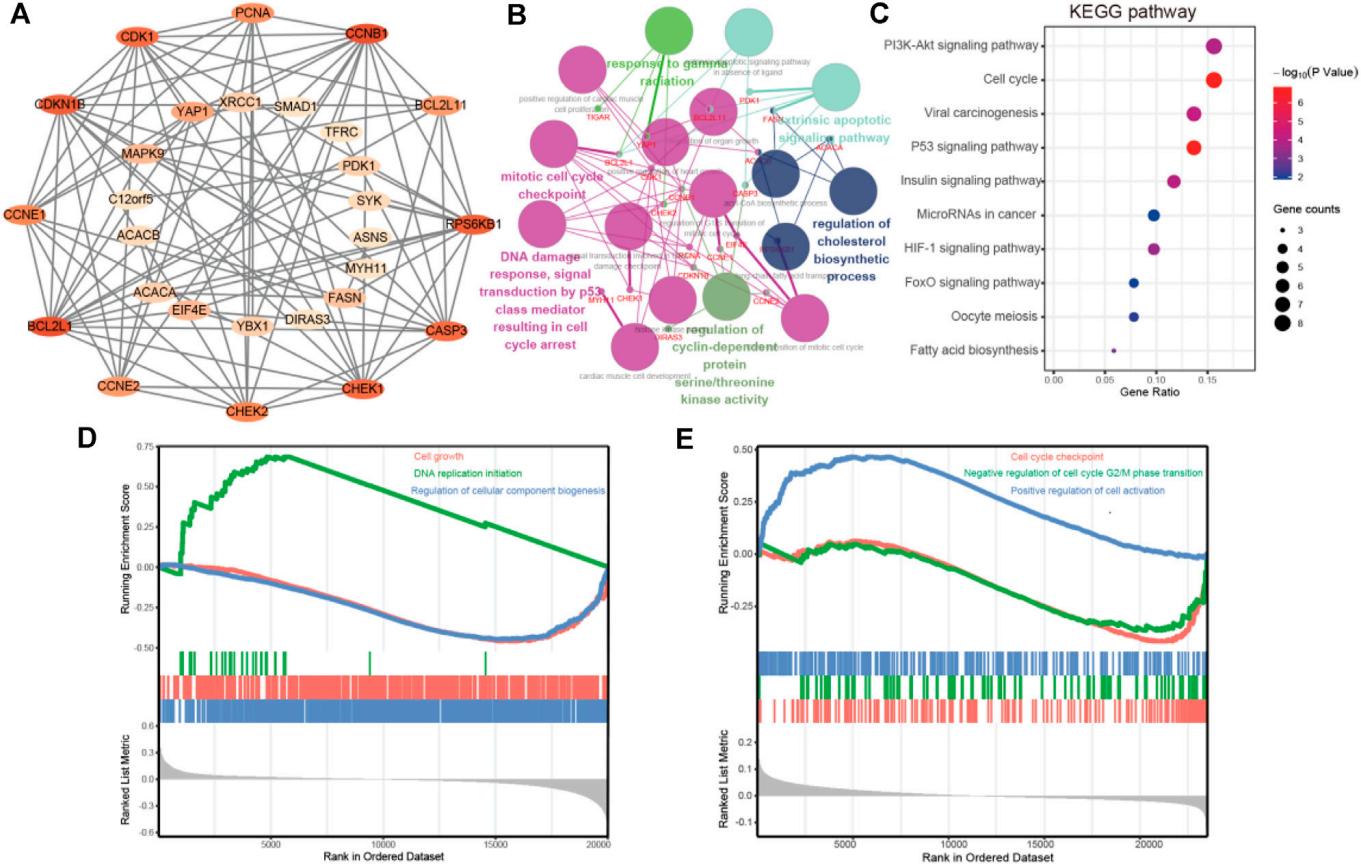
PPI network construction and enrichment analysis of FATP5 correlation genes. **(A)** Clustering analysis of FATP5 co-expressed genes by STRING tools. **(B)** The biological process analysis of hub genes was performed using the BiNGO plug-in, *p < 0.05. **(C)** KEGG pathway enrichment analysis of hub genes. GSEA enrichment analysis of FATP5 based TCGA dataset **(D)** and GSE39582 accession **(E)**.

The cBioPortal dataset was applied to obtain 29 genes in protein expression that are correlated with FATP5 in mRNA expression (*p*-value <0.05) for colorectal adenocarcinoma (TCGA, Firehose Legacy). We employed the STRING platform and cytoscape software to construct a protein-protein interact diagram of 29 co-expression genes (Figure 5A). In this PPI network, number of nodes:29, number of edges:89, average node degree:6.14, avg. local clustering coefficient:0.716, expected number of edges:25, PPI enrichment *p*-value:< 1.0e-16, these shown that co-expressed genes were closely related and highly reliable. Then, we made use of ClueGO and CluePedia to further performed the biological process analysis of hub genes. Cyclin-dependent protein serine/threonine kinase regulator activity, DNA damage response, signal transduction by p53 class mediator resulting in cell cycle and mitotic cell cycle checkpoint were largely altered (Figure 5B).

### GO and KEGG Pathway Enrichment Analysis Revealing Related Biological Functions of FATP5

Subsequently, we used the DAVID database for GO and KEGG pathway enrichment analysis to analysis biological functions of co-expression genes. A total of 46 enrichment results were obtained, including 21 biological processes, 5 molecular functions, and 5 cellular components, the top 5 go enrichment results of which were shown in Table 4. The biological processes mainly contained G1/S transition of mitotic cell cycle, positive regulation of cell proliferation, negative regulation of apoptotic process, cell proliferation, and positive regulation of mitotic cell cycle, which are consistent with the enrichment results in molecular functions and cellular components. Besides, KEGG pathway enrichment analysis shown that more genes were enriched in PI3K-AKT signaling pathway and cell cycle (Figure 5C). Based on TCGA dataset and GSE39582 accession, the enrichment results of GSEA demonstrate that FATP5 is closely related to the cell growth and cell cycle. In short, the enrichment analysis demonstrates that FATP5 plays an important role in regulating cell cycle in CRC.

**TABLE 4:** The top 5 terms of GO enrichment analysis results of co-expressed genes with FATP5.

Term	Description	Count in gene set	*p*-value
Molecular function			
GO:0005524	ATP binding	9	3.08E-03
GO:0016301	Kinase activity	3	3.51E-02
GO:0016538	Cyclin-dependent protein serine/threonine kinase regulator activity	2	2.64E-02
GO:0004075	Biotin carboxylase activity	2	1.11E-02
GO:0003989	Acetyl-CoA carboxylase activity	2	4.45E-03
Cellular component			
GO:0005634	Nucleus	12	4.83E-03
GO:0005829	Cytosol	8	9.20E-04
GO:0005739	Mitochondrion	6	1.75E-02
GO:0005813	Centrosome	5	2.28E-03
GO:0000781	Chromosome, telomeric region	2	3.86E-02
Biological pathway			
GO:0000082	G1/S transition of mitotic cell cycle	5	1.32E-06
GO:0008284	Positive regulation of cell proliferation	4	1.32E-02
GO:0043066	Negative regulation of apoptotic process	4	9.53E-03
GO:0008283	Cell proliferation	4	2.09E-03
GO:0045931	Positive regulation of mitotic cell cycle	4	1.49E-05

### siFATP5 Facilitates Cell Growth and Cell Cycle G2/M Phase Transition

**FIGURE 6:**
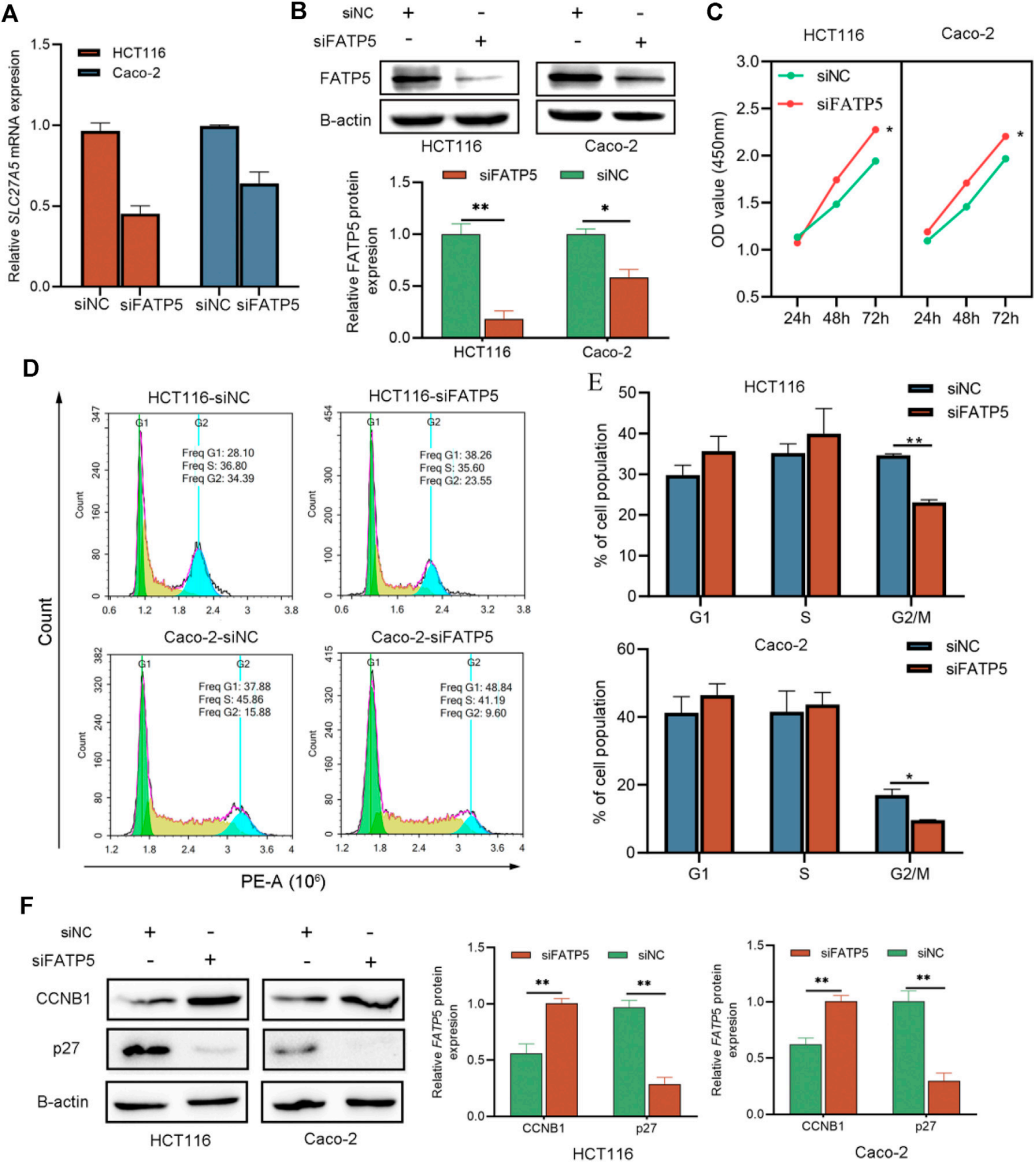
siFATP5 facilitates cell growth and cell cycle G2/M phase transition. FATP5 expression was detected after transfection in CRC cells by qRT-PCR **(A)** and western blotting **(B,C)** CCK-8 assays were used to detect cell viability of CRC cells after transfection. **(D,E)** Flow cytometric analysis was used to detect cell cycle distribution of CRC cells after transfection. **(F)** The expression of cycle-related protein. *p < 0.05, **p < 0.01.

To explore the effects of FATP5 on CRC cells, siRNA transfection technology was applied on CACO-2 and HCT116 cells, respectively. Transfection results were confirmed by qRT-PCR and western blotting, which indicate that siRNA had a good knockdown efficiency (Figures 6A,B). Then, we studied the effect of FATP5 on CRC cell proliferation *in vitro*. The cytotoxicity CCK-8 assay revealed that downregulation of FATP5 in both cell types significantly enhanced cell proliferation compared to that in the control cells (*p* < 0.05, Figure 6C). In addition, the results of flow cytometry also showed that the low expression of FATP5 had an obvious effect on cell cycle G2/M transition. Compared with the control group, the proportion of G2/M phase in siFATP5 group was significantly reduced (*p* < 0.05, Figures 6D,E). We also determined cycle-related proteins, showing that siFATP5 augmented CCNB1 expression while reduced p27 expression (*p* < 0.05, Figure 6F).

### Colorectal Tumors From Patients Have Higher FATP5 Expression Than Paracancerous Tissues

**FIGURE 7:**
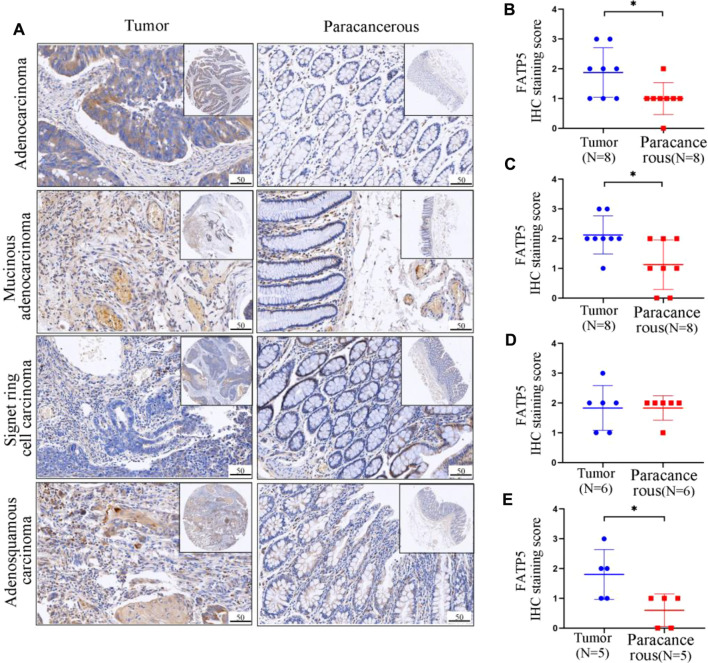
Representative IHC staining and quantitative analysis showing the expression of FATP5 in colorectal tumors and adjacent tumor tissues. **(A)** IHC staining of tumour tissues and paracancerous tissues. The protein expression levels of FATP5 in adenocarcinoma **(B)**, Mucinous. adenocarcinoma **(C)**, Signet ring cell carcinoma **(D)** and Adenosquamous carcinoma **(E)**. **p < 0.01.

Through mining the database, we found that the expression of FATP5 protein in colorectal cancer is significantly higher than that in normal tissues, and FATP5 is closely related to the prognosis of colorectal cancer patients. In order to confirm aforementioned findings, we analyzed the FATP5 expression in primary tissues of CRC patients and paracancerous tissues by IHC staining, including four pathological types (Figure 7A). FATP5 were located in cytoplasm and distributed diffusely. The results showed that, except in signet ring cell carcinoma (*n* = 6), the expression of FATP5 in adenocarcinoma (*n* = 8), mucinous adenocarcinoma (*n* = 8) and adenosquamous carcinoma (*n* = 5) was significantly higher than paracancerous tissues, which verified that FATP5 plays an important role in colorectal cancer (Figures 7B–E).

## Discussion

CRC is the fourth most common cancer and the second leading cause of cancer death in the United States (Benson et al., 2018). Besides, the challenges in treatment of this disease have not been fully addressed. Due to a paradigm shift in CRC risk factors, cancer prevention and early diagnosis, as well as treatment methods, CRC morbidity and mortality have partially improved. The pathogenesis and diagnosis of CRC remain an area of complexity, which needs further investigation (Auyeung and Ko, 2017). Studies have shown that early screening of diseases based on biomarkers can greatly reduce the mortality of diseases and improve the prognosis, which indicates that biomarkers play a vital role in the diagnosis and treatment of diseases (Wolf et al., 2018).

Changes in lipid metabolism contribute to different aspects of tumorigenesis and fatty acid abnormality is a risk factor of many tumors, including CRC (May-Wilson et al., 2017). FATPs play a role in the uptake and metabolism regulation of long-chain fatty acids, whose dysregulation has been reported in many cancers. Even though the role of FATPs activators in the tumorigenesis and prognosis of several cancers has been partially confirmed, further bioinformatics analysis of FATPs still remains to be conducted. FATPs also play a key character in many diseases, such as obesity, diabetes, cardiopathies, skin syndromes and carcinoma (Dourlen et al., 2015). During the progress of these diseases, FATP5 is involved in fatty acid transport and bile acid metabolism, which may be a crucial regulator in tumors. To our knowledge, our study explored the mRNA expression and prognostic values of FATP5 factors in CRC for the first time. We hope that our findings will complement previous research and contribute to existing knowledge, treatment designs improvement, and enhancing prognosis accuracy for CRC patients.

Molecular biomarkers that can be used as prognostic indicators many help determine the patient’s personalized treatment plan. In this study, we applied TCGA and GEO database to investigating the expression pattern of FATP5 in certain cancer types. The results revealed that FATP5 was the only overexpressed member of FATPs family in CRC tissues, implying its predominant role in regulating FA transport and activation in colorectal carcinoma. Besides, we found that FATP5 expression was higher and closely related to prognosis of CRC patients. Four independent data sets showed that the expression of FATP5 was closely related to OS in CRC patients. In addition, high-expression FATP5 and low-expression groups shown an obvious statistics differences in lymph node metastasis and distant metastasis, which indicated that FATP5 can be used to predict whether lymph node metastasis and lymph node metastasis occur in colorectal patients. Interestingly, although FATP5 was high expression in the tumor, the high expression level predicted a good prognosis, indicating that the expression and distribution of FATP5 and its receptors may interfere with tumor microenvironment, further affecting the prognosis. The research on the mechanism of FATP5 in the occurrence and development of CRC can help us to have a clear understanding of CRC, which can be used for clinical drug guidance and the research and development of targeted drugs. A similar phenomenon was also observed in CLDN4 expression and its prognostic significance in human pancreatic ductal adenocarcinoma. Higher CLDN4 expression represents better outcomes in human pancreatic ductal adenocarcinoma (Tsutsumi et al., 2012). These results highlighted that the prognostic value and clinical significance of FATP5 in CRC. Further research focusing on the prognosis relevance of FATP5 in larger prospective studies might also prove beneficial.

It has been revealed that FATP5 participated in PI3K/AKT signaling pathway (Liu et al., 2019), Cyclin-dependent protein serine/threonine kinase regulator activity and p53 signaling pathway (Liu et al., 2020), indicating FATP5 acted as a significant part to cell cycle in CRC. Previous study has shown that the PI3K/Akt signaling pathway and p53 signaling pathway are involved in cell proliferation and apoptosis in breast cancer (Chen et al., 2018), indicating the significant role of FATP5 in cell cycle. In addition, FA are the building blocks for all lipids and hence are essential components of all biological membrane structures. As a result, proliferating cells need to gain sufficient fatty acids to support membrane growth and integrity. Previous study has shown that *de novo* fatty acid synthesis serves as a vital factor in the intracellular fatty acid pool of tumor cells (Menendez and Lupu, 2007), implying the crucial role of FATP5 in cell cycle. Mechanistically, FATP5 is high expression in tumor cells, which can participate in the regulation of cell cycle by regulating lipid synthesis (Zhu and Thompson, 2019). Based on TCGA dataset and GSE39582 accession, the enrichment results of GSEA revealed that the high expression of FATP5 was associated with the negative regulation of cell cycle G2/M transition, which may explain the suggestion that FATP5 high expression represents a good prognosis in CRC. The results of flow cytometry also proved that the high expression of FATP5 had an obvious negative effect on cell cycle G2/M transition.

Previous study has also suggested that hepatic FATP5 expression is related to histological progression and loss of hepatic fat in Nonalcoholic Fatty Liver Disease (NAFLD) patients (Enooku et al., 2020), indicating that down-regulated expression of hepatic FATP5 in NAFLD is related to histological progression, and may be associated with hepatic fat loss during NASH progression to cirrhosis. Besides, FATP5 deficiency activates nrf2/txnrd1 pathway by increasing lipid peroxidation in HCC (Gao et al., 2020), suggesting that FATP5 acts as a novel tumor suppressor in the development of liver cancer and is positive correlation with the prognosis of HCC patients. These findings keep consistent with our results in CRC, further highlighting the prognostic relevance of FATP5 in a wide variety of human cancers.

## Conclusion

In summary, we have shown the up-regulation of FATP5 mRNA expression in CRC and verified its significance as prognostic factor. We supposed that up-regulation of FATP5 would predict prognosis in CRC more accurately, suggesting the crucial role of FATP5 as a novel diagnostic biomarker in this aggressive carcinoma, by regulating the cell cycle G2/M transition.

## Data Availability

The original contributions presented in the study are included in the article/Supplementary Material, further inquiries can be directed to the corresponding author.
